# Characterizing Tuberculosis Medication Side Effects Using Video Directly Observed Therapy Monitoring in Uganda: Opportunities for Digital Pharmacovigilance

**DOI:** 10.21203/rs.3.rs-10595035/v1

**Published:** 2026-08-06

**Authors:** Juliet N. Sekandi, Kelvin Bwambale, Alif Indiralarasati, Doreen K. Kalembe, Kisha Patel, Damalie Nakkonde, Sarah Zalwango

**Affiliations:** University of Georgia; University of Georgia; University of Georgia; University of Georgia; University of Georgia; Makerere University; Kampala Capital City Authority

**Keywords:** tuberculosis, pharmacovigilance, side effects, video directly observed therapy, VDOT, Uganda

## Abstract

**Background:**

Monitoring medication-related side effects is essential to tuberculosis (TB) pharmacovigilance, yet remains challenging in resource-limited settings. Video directly observed therapy (VDOT) offers an opportunity to capture patient-reported side effects in real time. This study characterized TB medication side effects reported through VDOT.

**Methods:**

We conducted a secondary analysis of 68 adults treated for drug-susceptible TB in a randomized VDOT trial in Kampala, Uganda, during 2020–2021. Side effects were recorded longitudinally through the VDOT platform. A severity-weighted burden score was developed from reporting frequency and severity, and latent class analysis identified burden subgroups.

**Results:**

Median age was 30 years; 50% were female and 31% were living with HIV. The most frequent side effects were joint/body pain (84%), body weakness (79%), and red/discolored urine (68%). Higher burden scores were observed among participants aged > 30 years (p = 0.030), people living with HIV (p = 0.023), smokers (p = 0.023), and those with lower TB knowledge (p = 0.033). Two subgroups emerged: High (66.2%) and Low Burden (33.8%).

**Conclusions:**

VDOT enabled longitudinal monitoring of medication-related side effects and revealed substantial variation in burden, along with associated factors. VDOT shows promise as a digital pharmacovigilance tool supporting patient-centered TB care. Future studies should evaluate its integration into routine TB programs.

## Introduction

Tuberculosis (TB) remains a major global public health challenge despite being a preventable and curable disease [1]. In 2024, an estimated 10.8 million people developed TB worldwide, and the disease continues to be a leading cause of death from a single infectious agent [2]. Tuberculosis treatment relies on prolonged multidrug regimens that are frequently associated with adverse drug reactions ranging from mild gastrointestinal symptoms to severe hepatotoxicity and neuropathy [3]. These events may lower treatment adherence and contribute to the development of drug-resistant TB, which requires longer, more toxic, and more costly regimens [4]. However, in many resource-limited settings, adverse events are often underreported or identified only during scheduled clinic visits, limiting opportunities for timely intervention [5, 6].

Video directly observed therapy (VDOT) has emerged as a promising digital tool for supporting TB treatment delivery and monitoring, compared to conventional DOT [7]. While VDOT has primarily been evaluated as an adherence-support intervention [8], its ability to capture repeated patient-reported symptoms throughout treatment presents an opportunity to strengthen pharmacovigilance efforts. Unlike conventional monitoring approaches that often rely on cross-sectional assessments, VDOT can generate longitudinal data on symptom occurrence, severity, and persistence in real time.

Previous studies have examined the prevalence and predictors of TB medication side effects [9–12]. However, less is known about the cumulative burden and reporting patterns of symptoms over the course of treatment. In part because adverse events are typically summarized as simple counts or binary indicators of occurrence, an approach that treats all side effects as equivalent regardless of how long they persist or how clinically severe they are. This limitation has been well documented in the broader pharmacological and clinical trials literature, where composite burden measure incorporating both duration and severity have been proposed as more clinically meaningful alternative to unweighted even counts [13]. Few studies have applied this kind of severity-weighted approach to TB pharmacovigilance data, and it remains unclear how frequently patients report side effects over time or whether distinct symptom burden profiles can be identified using longitudinal patient-reported data. Addressing these gaps may provide a more comprehensive understanding of patients’ treatment experiences and inform strategies for adverse event monitoring in routine TB care.

Using longitudinal symptom data collected through VDOT, this study characterized the prevalence, frequency, severity, and patterns of medication-related side effects among patients receiving treatment for drug-susceptible TB in Kampala, Uganda. We also identified patient characteristics associated with greater symptom burden and explored the potential utility of VDOT as a digital pharmacovigilance tool for TB care.

## Methods

### Study design and setting

This study was a secondary analysis of data from the DOT Selfie randomized controlled trial (RCT). The trial was conducted in five TB clinics across healthcare facilities in Kampala, Uganda, between July 2020 and October 2021. The study sites included Lubaga Private Not-for-Profit (PNFP) Hospital, Mulago National Referral Hospital, Kawaala Health Centre IV (HCIV), Kitebi HCIV, and Kisenyi HCIV.

### Participants

The study included all 68 people with TB assigned to the VDOT arm of the DOT Selfie RCT. Eligibility was based on the inclusion and exclusion criteria of the parent study, and the details of the methods are published elsewhere [8].

### Inclusion criteria

During the DOT Selfie study, participants were required to have a confirmed TB diagnosis, be within the first month of treatment, be aged 18 to 65 years, plan to reside in Kampala throughout their treatment, provide signed informed consent, and be proficient in Luganda or English.

### Exclusion criteria

Patients with TB were excluded if they had confirmed multidrug-resistant (MDR) or extensively drug-resistant (XDR) TB, were too ill to participate in study procedures lasting up to two hours, had cognitive, visual, or motor impairments preventing video recording, lacked access to electricity to charge a smartphone, or lived in areas without cellular network coverage.

### Sampling and sample size

Sixty-eight participants in the VDOT intervention arm were included in this secondary analysis. In the DOT Selfie RCT, participants were recruited using a consecutive sampling technique, enrolling every eligible TB patient until the target sample size was reached.

### Data collection

Data collected during the DOT Selfie RCT was used. At baseline, trained research assistants conducted interviews using a structured, interviewer-administered questionnaire as detailed in the published protocol [8]. The reported side effects were tracked through the Health Insurance Portability and Accountability Act (HIPAA)-compliant VDOT system, a secure cloud-based platform where submitted videos were stored. This tracking was performed by a trained study nurse.

### Variables

#### Derivation of the Severity-Weighted Side Effect Burden Score

To summarise each patient’s adverse-effect experience in a single measure, we constructed a Severity-Weighted Side Effect Burden Score. Simple adverse event counts treat all side effects as equivalent regardless of severity or duration, a well-documented limitation [13, 14]; the burden score addresses this by combining frequency and severity into one value.

Severity weights were assigned to each of the 15 side effects according to the Common Terminology Criteria for Adverse Events (CTCAE) v5.0, using a three-tier scheme consistent with the framework described by [13]: Grade 1 (mild) effects were weighted 1 and comprised fever, dizziness, headache, red urine, anorexia, and increased appetite; Grade 2 (moderate) effects were weighted 2 and comprised gastrointestinal upset, joint/body pain, body weakness/malaise, chest pain, skin rash, and puffy face; and Grade 3 (severe) effects were weighted 3 and comprised jaundice, numbness, and pedal edema (Table 1). Grades were assigned a priori by the study team using published CTCAE v5.0 definitions, in which mild, moderate, and severe events are distinguished by functional impairment and required intervention [14, 15]. Because patients reported only the occurrence and duration of each symptom during VDOT sessions, each grade reflects the typical clinical severity of that side effect category rather than a patient-specific assessment.

For each patient, the burden score was the sum, across all 15 side effects, of the severity weight multiplied by reporting frequency (the number of times the side effect was reported across all VDOT sessions during follow-up):

Side Effect Burden Score=Σ(Severity Weight⊠×Reporting Frequency⊠),i=1to15

To account for varying follow-up, scores were standardized to 30 days (dividing by total follow-up days, then multiplying by 30). A higher score reflects a greater overall treatment-related burden, capturing both the breadth and frequency of adverse effects.

Medication adherence was measured objectively via VDOT, which enabled real-time remote observation of ingestion by trained healthcare workers. Adherence was calculated as confirmed ingestion events divided by expected VDOT sessions over follow-up, expressed as a percentage.

TB-related knowledge was assessed using a 36-item questionnaire that covered topics such as the cause, transmission, and prevention of TB. Each correct answer scored 1 point, while incorrect or missing answers scored 0. The total score ranged from 0 to 36. Participants were categorized based on their scores: low knowledge (≤64%, score 0–23), moderate knowledge (65–74%, score 24–26), and high knowledge (≥75%, score 27–36). The scale demonstrated acceptable internal consistency, with a Cronbach’s alpha of 0.6890.

Other independent variables included HIV status, sex, age, education level, marital status, employment status, estimated monthly income, alcohol use, smoking status, and TB treatment history. Alcohol use was assessed by asking participants how often they consumed alcoholic beverages. Response options included: (1) No, (2) Yes, but rarely (about once a month), (3) Yes, moderately (at least once a week), and (4) Yes, frequently (daily). Due to the small number of participants reporting weekly/daily drinking, the variable was dichotomized into no alcohol use (category 1) and any alcohol use (categories 2–4) for analysis.

#### Data analysis

Descriptive statistics were used to summarize participants’ characteristics. Categorical variables were presented as frequencies and percentages. Continuous variables were summarized using means and standard deviations or medians and interquartile ranges (IQR), depending on the distribution of the data. The Mann–Whitney U test and Kruskal–Wallis test were used to compare the median burden score of reported side effects across covariates.

To identify distinct patterns of side effect co-occurrence among TB patients on VDOT, latent class analysis (LCA) was conducted on 14 binary side effect indicators. Model fit statistics were evaluated across one to four class solutions. The two-class solution was selected as the optimal model based on the lowest Bayesian Information Criterion (BIC = 978.86), perfect entropy (1.000) indicating complete separation between classes, and clinically interpretable profiles putting the sample size (n=68) into consideration.

#### Bias

The use of routinely recorded digital data minimized recall bias.

#### Ethical considerations

The study received ethical approval from the Institutional Review Boards of the University of Georgia (IRB ID: PROJECT00000571), Makerere University (Protocol No. 756), and the Uganda National Council for Science and Technology. All participants provided written informed consent prior to enrollment. Participants were compensated a flat rate of USD 10 for time and transportation associated with study visits. The study was conducted in accordance with the ethical principles of the Declaration of Helsinki.

## Results

### Background characteristics of study participants

The sample was equally distributed by sex, with 34 males and 34 females (50% each). The median age was 30 years (IQR 25, 42), majority had secondary or tertiary education (41, 60%) and 21 participants (31%) were HIV positive (Table 1).

### Distribution of side effects

**Figure 1:**
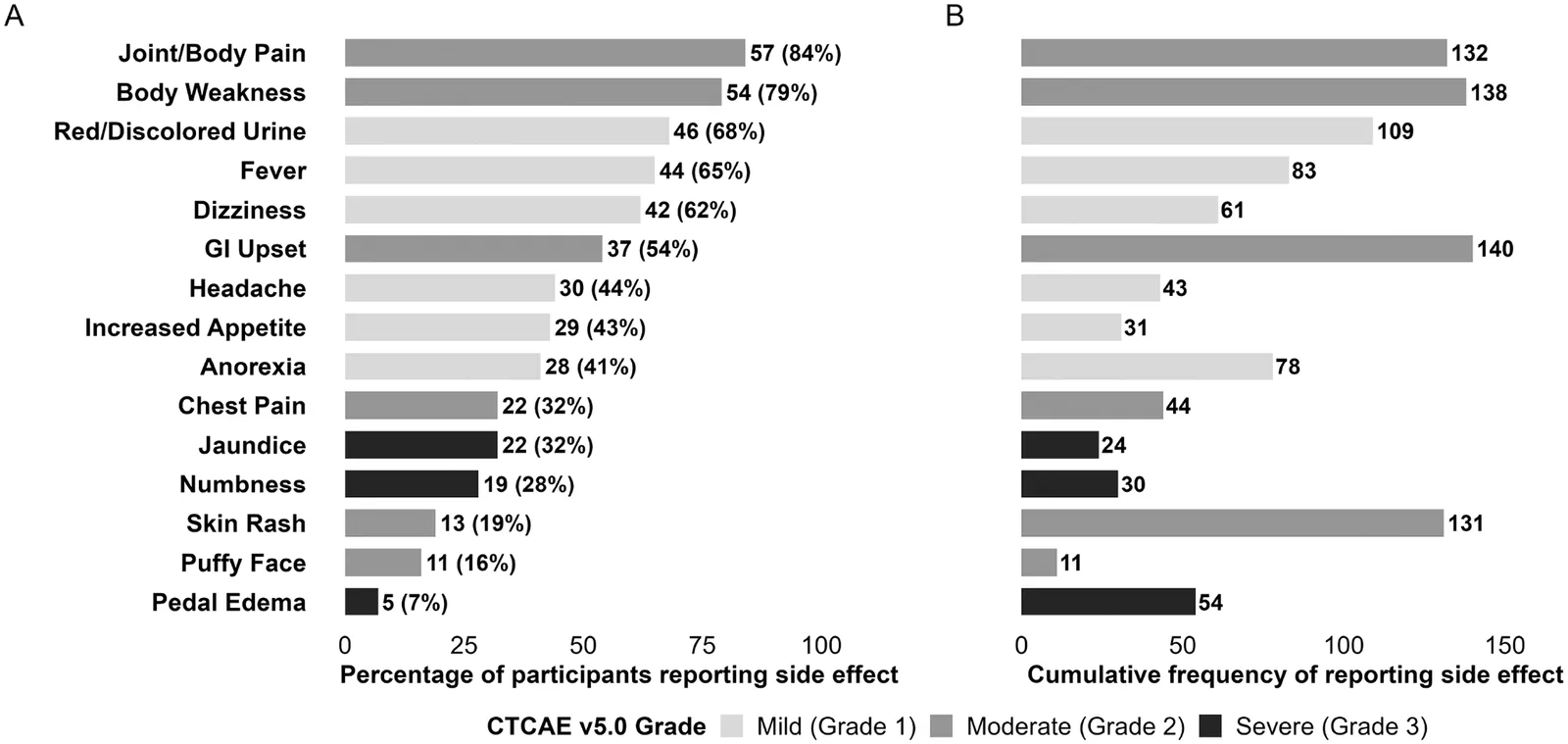
Panel A shows the percentage of patients reporting each side effect at least once. Panel B shows the cumulative number of times each side effect was reported across all patients. CTCAE = Common Terminology Criteria for Adverse Events v5.0 (NCI, 2017)

Joint/body pain was the most prevalent side effect (57, 84%), followed by body weakness (54, 79%) and red/discolored urine (46, 68%). Despite its relatively low prevalence (19%), skin rash had one of the highest cumulative frequencies (131 reports among 13 patients). Most side effects had a median reporting frequency of one (Figure 1).

### Distribution of Side Effect Burden Scores using the CTCAE v5.0 Grade

**Figure 2:**
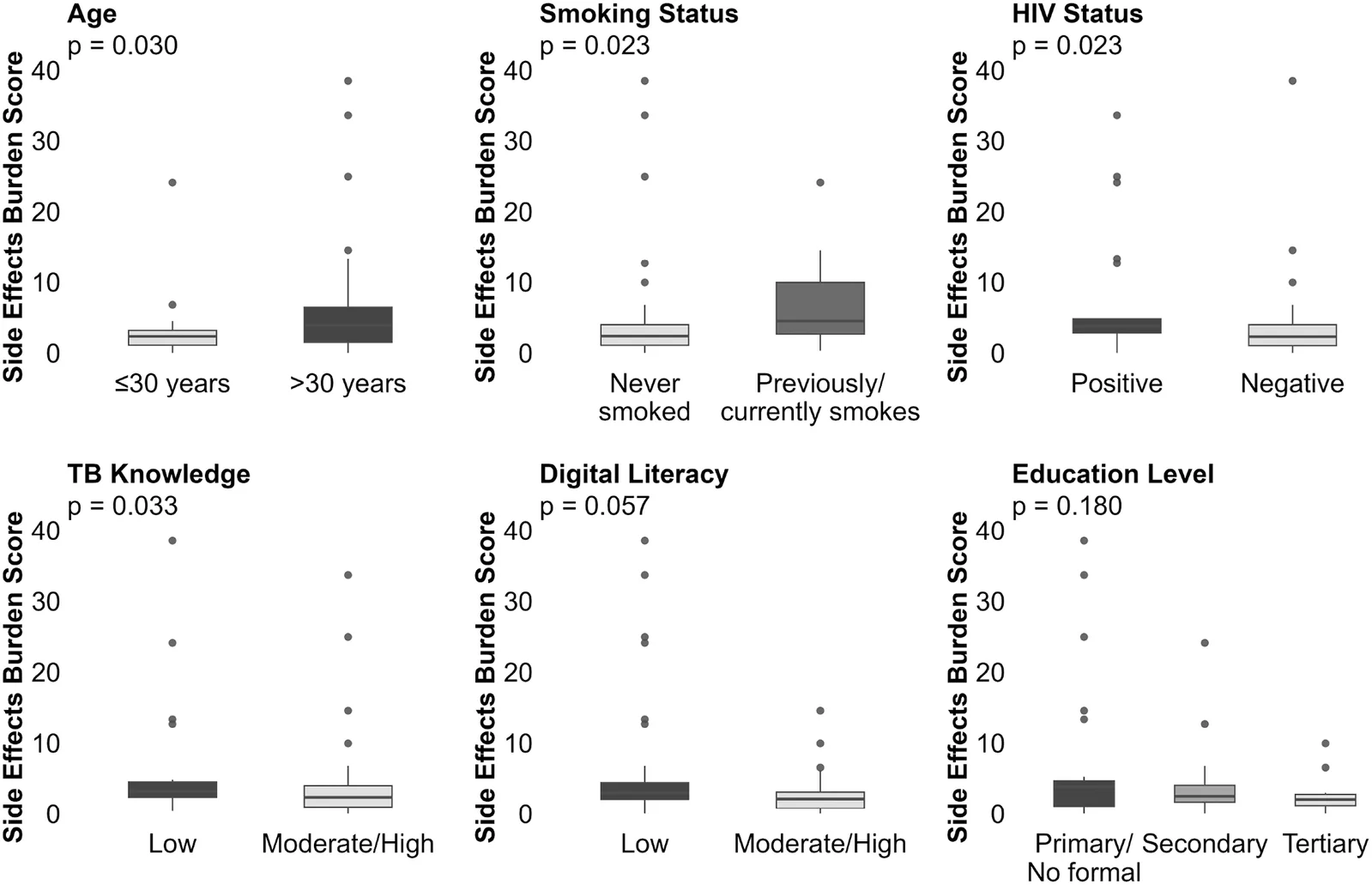
Box and whisker plots showing comparisons of side effect burden score by age category, smoking status, HIV status, TB knowledge, digital literacy, and education level (n=68). Box plots show median, IQR and outliers

The median side effect burden score differed significantly by age, smoking status, HIV status, and TB knowledge. Scores were higher in patients older than 30 years (3.89 [1.34, 6.53]) than in those aged ≤30 (2.33 [1.08, 3.25], p=0.030); in current or former smokers (4.50 [2.67, 9.93]) than never smokers (2.37 [1.08, 3.99], p=0.023); in people living with HIV (3.82 [2.83, 4.81]) than HIV-negative patients (2.29 [1.04, 3.98], p=0.023); and in those with low TB knowledge (3.17 [2.33, 4.50]) than those with moderate or high knowledge (2.33 [0.92, 3.98], p=0.033). Low digital literacy showed a borderline association with higher scores (3.00 [2.04, 4.40] vs 2.10 [0.76, 3.06], p=0.057) (Figure 2).

No significant differences were observed by sex (p=0.447), education (p=0.180), marital status (p=0.117), employment (p=0.515), monthly income (p=0.736), household size (p=0.562), alcohol use (p=0.499), or TB treatment history (p=0.445).

### Latent class analysis of side effect burden

The two-class solution identified a high burden class (Class 1, n=45, 66.2%) and a low burden class (Class 2, n=23, 33.8%). Table 2 presents the item-response probabilities for each side effect within each class. Class 1 was characterized by high probability of body weakness (1.000), joint pain (0.978), red urine (0.978), fever (0.956), and dizziness (0.889). Class 2 showed low item-response probabilities across all side effects. Notably, jaundice, numbness, headache, skin rash, and puffy face were also exclusively present in Class 1, with zero probability in Class 2 (Table 2).

### Latent class characteristics and adherence

The two classes differed markedly in side effect burden score, with Class 1 having a substantially higher median burden score (3.82 [IQR 2.50, 4.81]) compared to Class 2 (0.56 [IQR 0.09, 1.10], p<0.001). Males were disproportionately represented in the high burden class (60%) while females were more in the low burden class (70%), and this difference was statistically significant (p=0.039). Medication adherence did not differ significantly between the two classes (p=0.263). HIV positive status was more prevalent in Class 1 (38%) than in Class 2 (17%), though this difference did not reach statistical significance (p=0.103) (Table 3).

## Discussion

This study aimed to characterize medication-related side effects reported through VDOT among patients receiving treatment for drug-susceptible TB in Kampala, Uganda. To our knowledge, this is among the first studies to use longitudinal symptom data collected through VDOT to describe the prevalence, frequency, and burden of TB medication side effects in a resource-limited setting. Using repeated symptom reports collected throughout therapy, we identified substantial variation in side-effects burden among patients receiving treatment for drug-susceptible TB in Uganda.

We found that side effects were common, with joint/body pain, body weakness, and red/discolored urine reported by most participants (84%, 79%, and 68%, respectively). Beyond documenting prevalence, VDOT enabled repeated symptoms reported throughout treatment, revealing substantial heterogeneity in patient experiences. Two distinct side-effects burden subgroups emerged, characterized by high and low burden. Older age, HIV infection, smoking, and lower TB knowledge were associated with greater side-effects burden, although medication adherence remained high across both subgroups.

Previous studies of adverse drug reactions during TB treatment have largely focused on prevalence estimates or the occurrence of specific adverse events [9–12, 16]. While informative, these measures provide limited insight into how side-effects evolve throughout treatment. Our findings demonstrate the value of longitudinal symptom monitoring by showing that some side-effects were repeatedly reported despite relatively low prevalence. For example, skin rash and pedal edema were uncommon (19% and 7%, respectively) but generated disproportionately high reporting frequencies (131 and 54 reports, respectively). This suggests that prevalence alone may underestimate patients’ symptom burden and supports incorporating repeated symptom measurements into TB pharmacovigilance.

Participants living with HIV experienced greater side-effects burden, potentially reflecting overlapping toxicities from concomitant therapies and increased vulnerability to treatment-related side effects [17]. Older age, smoking, and lower TB knowledge were also associated with higher burden, consistent with previous literatures [11, 18, 19]. Males were more likely to belong to the high-burden class than females, although this finding should be interpreted cautiously given the small sample size.

Our findings have important implications for TB programs and policymakers. Although VDOT has primarily been evaluated as an adherence-support intervention, our results suggest that it can also serve as platforms for real-time pharmacovigilance by capturing symptom frequency, persistence, and burden that may not be routinely available through conventional monitoring systems. Integrating structured symptom reporting into VDOT could strengthen pharmacovigilance, particularly in resource-limited settings by enabling earlier identification of high-risk patients and more timely management of side effects. These findings may be especially relevant for MDR-TB programs, where treatment regimens are longer, more toxic, and associated with substantially higher rates of adverse events.

### Strength and limitations

This study has several notable strengths. To our knowledge, it is among the first studies to characterize medication-related side effects using longitudinal symptom data collected through VDOT in a resource-limited setting. Unlike conventional studies that primarily report the prevalence of adverse drug reactions, our analysis captured symptom frequency, persistence, and burden over time, providing a more comprehensive understanding of patients’ treatment experiences.

This study was a secondary analysis of a clinical trial with a relatively small sample size, which may limit statistical power and generalizability to routine TB care settings. In addition, symptom reports were self-reported through VDOT and were not independently clinically verified.

## Conclusion

VDOT enabled longitudinal characterization of TB medication side effects, demonstrating its potential to strengthen pharmacovigilance beyond traditional adherence monitoring. Integrating symptom surveillance into digital TB care platforms could improve patient safety and support more patient-centered TB treatment, particularly in resource-limited settings.

## Figures and Tables

**Table 1:** Study participant characteristics (n=68)

	Summary measure, N=68
Sex	
Female	34 (50%)
Male	34 (50%)
Age in completed years, [Median (Q1, Q3)]	30 (25, 42)
Age category	
≤ 30 years	38 (56%)
> 30 years	30 (44%)
Education level	
Primary or no formal education	27 (40%)
Secondary or tertiary education	41 (60%)
Marital status	
Never married	35 (51%)
Currently married	21 (31%)
Previously married	12 (18%)
Employment status	
Yes	41 (60%)
No	27 (40%)
Monthly Income, [Median (Q1, Q3)]	USD 53 (27, 128)
Smoking status	
Never smoked	57 (84%)
Previously/currently smokes	11 (16%)
Alcohol use	
No	58 (85%)
Yes	10 (15%)
TB treatment history	
Previous TB treatment	19 (28%)
No previous TB treatment	49 (72%)
HIV status	
Positive	21 (31%)
Negative	47 (69%)

**Table 2:** Item-response probabilities for each side effect by latent class

Side Effect	Class 1: High Burden (n=45)	Class 2: Low burden (n=23)
Joint/Body Pain	0.978	0.565
Jaundice	0.489	0.000
Red/Discolored Urine	0.978	0.087
Anorexia	0.556	0.130
Fever	0.956	0.043
Body Weakness	1.000	0.391
Increased Appetite	0.600	0.087
GI Upset	0.689	0.261
Headache	0.667	0.000
Skin Rash	0.289	0.000
Dizziness	0.889	0.087
Chest Pain	0.378	0.217
Numbness	0.422	0.000
Puffy Face	0.244	0.000

**Table 3:** Characteristics of latent classes (n=68)

	Class 1: High burden (n=45, 66.2%)	Class 2: Low burden (n=23, 33.8%)	P-value
**Sex**			
Male	27 (60%)	7 (30%)	**0.039**
Female	18 (40%)	16 (70%)	
**Side effect burden score, Median (Q1, Q3)**	3.82 (2.50, 4.81)	0.56 (0.09, 1.10)	**<0.001**
**Medication Adherence, Median % (Q1, Q3)**	93.8 (83.3, 98.3)	98.1 (92.6, 98.8)	0.263
**HIV Status, n (%)**			
Positive	17 (38%)	4 (17%)	0.103
Negative	28 (62%)	19 (83%)	

## Data Availability

The data of this study are available from the corresponding author upon reasonable request.
